# Validation of 2006 WHO Prediction Scores for True HIV Infection in Children Less than 18 Months with a Positive Serological HIV Test

**DOI:** 10.1371/journal.pone.0005312

**Published:** 2009-04-24

**Authors:** Cécile Alexandra Peltier, Christine Omes, Patrick Cyaga Ndimubanzi, Gilles François Ndayisaba, Sara Stulac, Vic Arendt, Olivier Courteille, Narcisse Muganga, Kizito Kayumba, Jef Van den Ende

**Affiliations:** 1 Esther Project/Luxembourg (Lux-Development), Kigali, Rwanda; 2 Rwinkwavu Hospital, Partners in Health, Rwinkwavu, Rwanda; 3 Treatment and Research AIDS Center/Center for Infectious Disease Control, Kigali, Rwanda; 4 Department of Pediatrics, University Teaching Hospital, Kigali, Rwanda; 5 Department of Infectious Diseases, CHU Luxembourg, Luxembourg, Luxembourg; 6 Institute of Tropical Medicine, Antwerp, Belgium; 7 University Hospital Antwerp, Antwerp, Belgium; University of Stellenbosch, South Africa

## Abstract

**Introduction:**

All infants born to HIV-positive mothers have maternal HIV antibodies, sometimes persistent for 18 months. When Polymerase Chain Reaction (PCR) is not available, August 2006 World Health Organization (WHO) recommendations suggest that clinical criteria may be used for starting antiretroviral treatment (ART) in HIV seropositive children <18 months. Predictors are at least two out of sepsis, severe pneumonia and thrush, or any stage 4 defining clinical finding according to the WHO staging system.

**Methods and Results:**

From January 2005 to October 2006, we conducted a prospective study on 236 hospitalized children <18 months old with a positive HIV serological test at the national reference hospital in Kigali. The following data were collected: PCR, clinical signs and CD4 cell count.

Current proposed clinical criteria were present in 148 of 236 children (62.7%) and in 95 of 124 infected children, resulting in 76.6% sensitivity and 52.7% specificity. For 87 children (59.0%), clinical diagnosis was made based on severe unexplained malnutrition (stage 4 clinical WHO classification), of whom only 44 (50.5%) were PCR positive. Low CD4 count had a sensitivity of 55.6% and a specificity of 78.5%.

**Conclusion:**

As PCR is not yet widely available, clinical diagnosis is often necessary, but these criteria have poor specificity and therefore have limited use for HIV diagnosis. Unexplained malnutrition is not clearly enough defined in WHO recommendations. Extra pulmonary tuberculosis (TB), almost impossible to prove in young children, may often be the cause of malnutrition, especially in HIV-affected families more often exposed to TB. Food supplementation and TB treatment should be initiated before starting ART in children who are staged based only on severe malnutrition.

## Introduction

Half of human immunodeficiency virus (HIV) infected children in resource limited countries will die before 2 years of age if antiretroviral treatment (ART) is not available [1]. Without polymerase chain reaction (PCR), early diagnosis of HIV in young children is a real challenge, as all infants born to HIV positive mothers have maternal HIV antibodies crossing the placental barrier, sometimes persisting up to the age of 18 months [2]. Definitive diagnosis of HIV infection is only possible using advanced technical approaches such as PCR to confirm presence of the virus in the blood [3]. In recent years, HIV PCR (DNA or RNA) has become more accessible for resource limited countries: blood specimen sampling, storage and transport have been simplified with the use of dried blood spot [4] and prices are decreasing with the introduction of real time PCR [5]. However, the equipment remains expensive and requires highly trained laboratory technicians. Other diagnostic techniques are being developed, such as detection of immune-complex dissociated P24, but have not been widely applied or validated [6]. In the immediate future, HIV PCR will not be available throughout most resource limited countries. The possibility of treating children having positive clinical signs and a positive HIV serological test, without the obligation of doing PCR is an important opportunity [7], preventing children from dying in places where ART is already available and free of charge, but where diagnostic PCR is not. These problems primarily affect African countries, and 420 000 children are newly infected every year worldwide [8].

In December 2004, the World Health Organisation (WHO) first proposed using clinical criteria to diagnose HIV infection and to recommend ART for children under 18 months old, first as Web-based drafts, and finally reported as the 2005 WHO clinical staging system [9]. The criteria proposed considering a child less than 18 months old infected if a HIV serological test was positive (indicating either exposure or infection) and if two of the following clinical signs were present: sepsis, severe pneumonia, thrush or severe malnutrition. At the Toronto 2006 AIDS conference, a sensitivity of 68.8% and a specificity of 74.1% for HIV infection were reported using these criteria [10].

Criteria were slightly modified in the latest paediatric WHO guidelines published in August 2006 [11] after the Toronto conference, but specificity and sensitivity have not yet been evaluated and were not modified by the latest WHO technical group which met in Geneva in April 2008 [12]. At this meeting, an important decision was made to initiate ART for all HIV infected or clinically suspected (according to the criteria evaluated in this article) children under the age of one year.

These 2006 criteria suggest that ART should be initiated if HIV seropositive children <18 months have two or more of the following clinical signs: sepsis, severe pneumonia, thrush or any WHO stage 4 clinical signs (Box S1).

Immune depression indicated by CD4 <25% for children ≤11 months old, and CD4 <20% for children between 12 and 18 months is also a factor for diagnosis of HIV infection apart from clinical criteria [11].

This study intends to compare the latest WHO (August 2006) clinical and immunological criteria for HIV infection in children less than 18 months with previous criteria, as well as to evaluate the sensitivity and specificity of low CD4 cell counts for HIV diagnosis, all with HIV PCR (DNA) as the gold standard.

We also discuss recommendations to improve the specificity of these indispensable clinical scores, since universal access to PCR remains far from reality.

## Materials and Methods

In this prospective study we included all HIV seropositive children <18 months old admitted between January 2005 and December 2007 to the paediatric ward of the “Centre Hospitalier Universitaire de Kigali” (CHUK), a national reference teaching hospital in Rwanda. Serostatus was analyzed with a first positive serological rapid test (First Response, PMC Medical, Daman, India), and confirmed with a second rapid test (Unigold, Trinity Biotech plc, Wicklow, Ireland). In the case of discordant results, a third rapid test (Capillus, Trinity Biotech plc, Wicklow, Ireland) was added. The following data were collected: DNA PCR (Amplicor version 1.5, Roche Molecular Systems, Pleasanton, CA), clinical signs, and absolute and/or relative CD4 cell count. In the case of lack of reagents or other logistical problems, blood was preserved in order to collect all data on a single day. Each child was considered only once, even if hospitalised several times.

Until July 2005, absolute CD4 cell counts were measured with a Cyflow counter (Partec, Munster, Germany). Relative CD4 cell counts were estimated by the following algorithm: CD4 <1500 = CD4 <25% for children ≤11 months old, and CD4 <750 = CD4 <20% for children between 12 and 18 months old [11].

Starting in August 2005, absolute and relative CD4 cell counts were done with FACSCalibur (BD Bioscience, Becton, Dickinson and company, USA).

Severe malnutrition was determined as weight for age ≤3 Z scores (WHO international weight and length charts [13] ) or weight for height <70% [14]. AIDS-defining opportunistic infections (stage 4) were prospectively recorded (Box S2). Sepsis was diagnosed by the presence of fever and at least two of the following clinical signs: pulse >180/minute, respiratory rate >40/minute, capillary refill >3 seconds. Oxygen, IV antibiotics and volume expansion were given. The diagnosis of severe pneumonia was based on chest X-ray and oxygen requirement. Definition of oral candidiasis (thrush) relied on clinical observation of white creamy small plaques in the mouth [11].

Clinical values and immunological results were compared to the results of PCR, which has reported 98% sensitivity and specificity for infected children after the age of four weeks [15]. For statistical analyses we relied on Microsoft Office Excel 2003 (Microsoft Corporation, Redmond, Washington) and SPSS (SPPS inc. 14.0.0, Chicago, Illinois).

We received ethical approval for this study from the Research Board of CHUK, recognized by the national ethical committee. Only interventional studies or studies requiring examinations not included in routine work-up would have required referral to the national ethics committee. All hospitalized children in Rwanda are offered routine HIV testing after counselling of parents or legal guardians; no written consent is performed. This corresponds to Rwanda's national recommendations for scaling-up of HIV testing of children. For collecting these data, no additional blood samples or extra physical examinations were performed. The analyses of these data are done with identification numbers corresponding to each patient to preserve patient anonymity.

## Results

Two hundred and thirty six children matched the study inclusion criteria; 105 children were female, 131 were male, and the median age was 9 months (20 days to 18 months old). 124 of 236 (52.5%) of children were confirmed to be infected with HIV using HIV DNA PCR (Table 1). Half of the children (123/236, 52.1%) had severe unexplained malnutrition corresponding to WHO clinical stage 4 (Box S2), whereas septicaemia, pneumonia or thrush were present in 28.0%, 42.0% and 36.0% respectively. Seventy nine percent of children were in WHO stage 3 or 4, and 39.4% had low CD4 cell counts (Table 1).

**Table 1:** Characteristics of 236 hospitalized children under 18 months old with an HIV-1 serological positive test in Rwanda.

Characteristics (N = 236)	Proportion or mean	%
Gender (M/F)	131/105	55.5/44.5
Average age	9 months (range 20 days-18 months)	
HIV PCR positive	124	52.5
WHO clinical stage I*	27	11.4
WHO clinical stage II	22	9.3
WHO clinical stage III	57	24.2
WHO clinical stage IV	130	55.1
Severe malnutrition	123	52.1
Septicemia	66	28
Thrush	85	36
Pneumonia	99	41.9
low CD4 cell count	93	39.4

* ref [11].

Logistic regression of predictors for HIV infection in children <18 months showed significance for WHO clinical criteria, CD4 cell counts and age, but not for sex (Table 2). One hundred and forty eight children (62.7%) displayed WHO 2006 clinical HIV-defining criteria, of which 87 (58.7%) met 2006 criteria based on unexplained severe malnutrition alone. Only 44 (50.5%) of these severely malnourished children were PCR positive.

**Table 2:** Logistic regression of individual predictors for HIV infection in children <18 months.

Variables in the Equation						
	B	S.E.	Wald	df	Sig.	Exp (B)
Age	0.127	0.036	12.627	1	0.000	1.135
Sex	−0.157	0.332	0.225	1	0.635	0.855
Malnutrition	0.386	0.186	4.299	1	0.038	1.471
Septicemia	0.956	0.381	6.290	1	0.012	2.600
Pneumonia	1.249	0.338	13.690	1	0.000	3.487
Thrush	1.518	0.363	17.485	1	0.000	4.564
CD4 cell count	1.402	0.347	16.351	1	0.000	4.065
Constant	−3.237	0.570	32.296	1	0.000	0.039

**Hosmer and Lemeshow:** 0.469.

Clinical 2006 WHO criteria had a sensitivity of 76.6% and a specificity of 52.7%.

Using 2005 interim WHO clinical criteria would have resulted in a sensitivity of 68.5% and a specificity of 71.4% (Table 3). Low CD4 cell counts had a sensitivity of 55.6% and a specificity of 78.5%, with a positive predictive value (PPV) of 74.2% and a negative predictive value (NPV) of 61.5%. Combining clinical criteria with CD4 cell count resulted in sensitivity of 88.7% and specificity of 42.9%, and combining them with age >12 months gave sensitivity of 83.9% and specificity of 45.5% (Table 3).

**Table 3:** Sensitivity and specificity of different WHO presumptive diagnostic alternatives of HIV infection in children <18 months with a positive serological test for HIV in Rwanda.

Criteria	Sensitivity (CI)	Specificity (CI)	PPV	NPV
Interim WHO 2005	68.5 (59.6–76.6)	71.4 (62.1–79.6)	72.6	67.2
WHO 2006	76.6 (68.2–83.7)	52.7 (43–62.2)	64.1	67
Low CD4 cell count	55.6 (46.5–64.6)	78.5 (69.8–85.8)	74.1	61.4
WHO 2006 plus age >12 months	83.9 (76.2–89.9)	45.5 (36.1–55.2)	63	71.8
WHO 2006 plus CD4 cell count	88.7(81.8–93.8)	42.9 (33.5–52.6)	63.2	77.4

CI: confidence intervals.

PPV: positive predictive value.

NPV: negative predictive value.

McNemar testing of differences in sensitivities and specificities showed significance for all paired comparisons.

## Discussion

This study evaluated the revised WHO algorithm for predicting HIV infection in children under 18 months old. Comparing the WHO 2005 interim clinical presumptive diagnosis guidelines with the August 2006 guidelines, the new guidelines gained in sensitivity but lost in specificity, mainly due to the inclusion of severe unexplained malnutrition as a stage 4-defining criterion, for which ART is recommended. More HIV uninfected children risk incorrectly receiving ART because of “unexplained malnutrition”. Many of these infants are also exposed to poverty and tuberculosis (TB), conditions that also lead to malnutrition [16]–[18] .

CD4 cell count and total lymphocyte count are known to have a low sensitivity for HIV infection; this was also observed for CD4 cell counts in our cohort. Low CD4 cell counts and low total lymphocyte counts are known markers of progression leading to mortality in resource–rich countries [19] and have already been evaluated as markers indicating need to start ART in resource limited countries [20] but have not been evaluated as markers of infection for children under 18 months old. We did not include the evaluation of low lymphocyte count in this study given the extremely low predictive value found hitherto. The method of extrapolation of CD4 cell percentage based on absolute CD4 cell count risks misclassification of children with lymphopenia or lymphocytosis. Severe malnutrition can also influence CD4 cell values and could have an effect on the predictive value of CD4 cell counts for HIV infection, as 52.1% of our children were severely malnourished.

Adding CD4 cell count to the WHO clinical criteria increases the sensitivity of HIV diagnosis, but lowers the specificity considerably. This study shows that an alternative: adding persistent anti-HIV antibodies at ages over 12 months as a predictor, also increases sensitivity and lowers specificity, but to a minor degree (Table 3).

Could the study's setting in a reference hospital have influenced the results, making them difficult to apply to other situations? The population of children hospitalized at a tertiary level is generally more seriously ill, increasing the sensitivity of the clinical scores. But university and other reference hospitals in developing countries almost always also serve as district hospitals for the city; only a minority of the patients was truly referred in 2005. In addition, the country's poverty could have had the same effect, but WHO guidelines were conceived for countries where PCR is not available, which are mainly poor to very poor settings.

One important confounding factor for clinical diagnosis of HIV in children is tuberculosis, as children born into HIV-affected families are more frequently exposed to TB [21]. Young children infected with TB can be stunted, with malnutrition-related immune depression clinical signs including sepsis, thrush and pneumonia, and may also be anergic to PPD [22]. Malnourished children also have severe cellular immunodeficiency [23].

Tuberculosis is extremely difficult to diagnose in young children [24], and disseminated and extra-pulmonary tuberculosis occur more frequently than in adults [25]. Mycobacterium blood culture, PCR, and bronchoalveolar lavage and cultures are rarely available in resource limited settings. Moreover, the yield of microscopic examination of gastric fluid samples for acid fast bacilli is low (5 to 10%) [26] even when advanced laboratory tests are available. Children with severe malnutrition do not present with classic inflammatory signs of tuberculosis infection [27] such as cough, fever, enlarged lymph nodes and positive X-rays [28]. A mortality rate of up to 40% for children co-infected with HIV and tuberculosis has been reported [29].

Clinical scores and empiric TB treatment are not described in the WHO definition for unexplained malnutrition. Without clearly defining a method for excluding TB, “unexplained” stunting will be overestimated. In countries where the epidemics of tuberculosis and HIV exist concurrently, confusion between the two diseases will continue to be a reality [30]. Treating HIV with ART without first initiating TB treatment in children harbouring both infections can lead to fulminant tuberculosis as part of immune reconstitution inflammatory syndrome. Worse, treating an HIV suspected but actually HIV negative child with ART bears the risk of overlooking TB as the true diagnosis [31].

Future research could evaluate the following strategy, in use in Rwanda since this study: Children under 18 months old with a positive HIV serological test, who meet WHO criteria for initiating ART based only on severe unexplained malnutrition, should be started for one month on food supplementation and empiric TB treatment. Initiation of ART could then be based on the child's response to these interventions to exclude malnutrition due to food insufficiency and TB.

Based on the results of the CHER study [32], WHO recently [12] recommended systematic ART for all infected or even clinically suspected children under 12 months if PCR is not available, irrespective of CD4 or clinical stage [32]. This study demonstrated lower overall mortality in the ART group.

As clinicians, we hope to be able to continue saving children's lives, even when lacking the technology for clinical diagnosis. We should evaluate the clinical response to food supplementation and empiric TB treatment, and validate clinical scoring using this algorithm, to improve the specificity of actual HIV clinical signs. In the meantime, scaling up access to HIV PCR testing is urgently needed.

Using revised August 2006 WHO criteria for diagnosis of HIV infection in children under 18 months results in a 53.6% specificity, 17.7% lower than the 2005 WHO clinical guidelines, merely due to the acceptance of unexplained severe malnutrition as a single criterion defining stage 4 HIV infection. Although 3/4 of infected children were detected by clinical criteria alone, we would have treated 46.4% of uninfected children without the availability of PCR. Beyond the cost and risks of treating uninfected children, this also may lead clinicians to fail to diagnose other existing conditions, especially TB.

The low diagnostic accuracy of these clinical criteria and of CD4 cell count emphasizes the urgent need for availability of HIV PCR testing in low resource countries and for better diagnostic tools for tuberculosis in young children.

## Supporting Information

Box S1 Reminder of latest WHO guidelines: Antiretroviral therapy of HIV infection in infants and children in resource-limited settings: toward universal access 2006 (Page 22).
(0.03 MB DOC)

Box S2 Reminder of latest WHO guidelines: Antiretroviral therapy of HIV infection in infants and children in resource-limited settings: toward universal access 2006 (Page 85).
(0.03 MB DOC)
