# Supplementary material for: Validation of 2006 WHO Prediction Scores for True HIV Infection in Children Less than 18 Months with a Positive Serological HIV Test
Source: PLoS One. 2009 Apr 24;4(4):e5312. doi: 10.1371/journal.pone.0005312 (PMC2669178; doi:10.1371/journal.pone.0005312)
Supplement: Box S1 — Reminder of latest WHO guidelines: Antiretroviral therapy of HIV infection in infants and children in resource-limited settings: toward universal access 2006 (Page 22). (0.03 MB DOC) [file pone.0005312.s001.doc]

**BOX S1: Reminder of latest WHO guidelines: Antiretroviral therapy of HIV infection in infants and children in resource-limited settings: toward universal access 2006 (Page 22).**

**Clinical criteria for presumptive diagnosis of severe HIV disease in infants and children aged under 18 months requiring ART in situations where virological testing is not available**

A presumptive diagnosis of severe HIV disease should be made if:

- The infant is confirmed as being HIV antibody-positive

**and**

- Diagnosis of any AIDS-indicating condition(s) can be made

**or**

- The infant is symptomatic with two or more of the following:

- Oral thrush;

- Severe pneumonia;

- Severe sepsis.

Other factors that support the diagnosis of severe HIV disease in an HIV-seropositive infant include:

- Recent HIV-related maternal death or advanced HIV disease in the mother;
- Percentage of CD4 cell count <20

Confirmation of the diagnosis of HIV infection should be sought as soon as possible.
