# Supplementary material for: Validation of 2006 WHO Prediction Scores for True HIV Infection in Children Less than 18 Months with a Positive Serological HIV Test
Source: PLoS One. 2009 Apr 24;4(4):e5312. doi: 10.1371/journal.pone.0005312 (PMC2669178; doi:10.1371/journal.pone.0005312)
Supplement: Box S2 — Reminder of latest WHO guidelines: Antiretroviral therapy of HIV infection in infants and children in resource-limited settings: toward universal access 2006 (Page 85). (0.03 MB DOC) [file pone.0005312.s002.doc]

**BOX S2: Reminder of latest WHO guidelines: Antiretroviral therapy of HIV infection in infants and children in resource-limited settings: toward universal access 2006 (Page 85).**

**Clinical stage 4(i) (ii)**

- Unexplained severe wasting, stunting or severe malnutrition not responding to standard therapy
- Pneumocystis pneumonia
- Recurrent severe bacterial infections (e.g. empyema, pyomyositis, bone or joint infection, meningitis)
- Chronic herpes simplex infection; (orolabial or cutaneous of more than one month’s duration)
- Extrapulmonary TB
- Kaposi sarcoma
- Oesophageal candidiasis (or Candida of trachea, bronchi or lungs)
- Central nervous system toxoplasmosis (after the neonatal period)
- HIV encephalopathy
- Cytomegalovirus (CMV) infection; retinitis or infection affecting another organ > 1 month of age
- Extrapulmonary cryptococcosis (including meningitis)
- Disseminated endemic mycosis (extrapulmonary histoplasmosis, coccidiomycosis)
- Chronic cryptosporidiosis (with diarrhoea )
- Chronic isosporiasis
- Disseminated non-tuberculous mycobacteria infection
- Cerebral or B cell non-Hodgkin lymphoma
- Progressive multifocal leukoencephalopathy
- HIV-associated cardiomyopathy or nephropathy

(i) Unexplained refers to where the condition is not explained by other causes.

(ii) Some additional specific conditions can be included in regional classifications
